# EGTA Can Inhibit Vesicular Release in the Nanodomain of Single Ca^2+^ Channels

**DOI:** 10.3389/fnsyn.2019.00026

**Published:** 2019-10-01

**Authors:** Yukihiro Nakamura

**Affiliations:** Department of Pharmacology, Jikei University School of Medicine, Tokyo, Japan

**Keywords:** Ca^2+^, Ca^2+^ channel, Ca^2+^ microdomain, reaction diffusion simulation, vesicular sensor, coupling distance, EGTA, transmitter release

## Abstract

The exogenous Ca^2+^ chelator EGTA (ethylene glycol tetraacetic acid) has been widely used to probe the coupling distance between Ca^2+^ channels and vesicular Ca^2+^ sensors for neurotransmitter release. Because of its slow forward rate for binding, EGTA is thought to not capture calcium ions in very proximity to a channel, whereas it does capture calcium ions at the remote distance. However, in this study, our reaction diffusion simulations (RDSs) of Ca^2+^ combined with a release calculation using vesicular sensor models indicate that a high concentration of EGTA decreases Ca^2+^ and vesicular release in the nanodomain of single channels. We found that a key determinant of the effect of EGTA on neurotransmitter release is the saturation of the vesicular sensor. When the sensor is saturated, the reduction in the Ca^2+^ concentration by EGTA is masked. By contrast, when the sensor is in a linear range, even a small reduction in Ca^2+^ by EGTA can decrease vesicular release. In proximity to a channel, the vesicular sensor is often saturated for a long voltage step, but not for a brief Ca^2+^ influx typically evoked by an action potential. Therefore, when EGTA is used as a diagnostic tool to probe the coupling distance, care must be taken regarding the presynaptic Ca^2+^ entry duration as well as the property of the vesicular Ca^2+^ sensor.

## Introduction

The release of neurotransmitter is triggered by an increase in the intracellular Ca^2+^ concentration ([Ca^2+^]_*i*_) in the presynaptic nerve terminal (Katz, 1969). This increase in [Ca^2+^]_*i*_ is mediated by voltage-gated calcium channels (VGCCs), activated upon the arrival of action potentials or by sustained depolarizations. Calcium ions entering through a channel pore diffuse to vesicular Ca^2+^ sensors. During this diffusion, part of calcium ions binds to Ca^2+^ buffers present in the cytosol. Thus, the probability of vesicular release, i.e., the output from the presynaptic nerve terminal, is determined by the combination of Ca^2+^ influx, the properties of Ca^2+^ buffers and Ca^2+^ sensors, and the distance between VGCCs and Ca^2+^ sensors. This coupling distance between VGCCs and Ca^2+^ sensors is an important determinant of the [Ca^2+^]_*i*_ sensed by the Ca^2+^ sensor, because the spatial concentration gradient of Ca^2+^ formed around the open channel is steep. This gradient was first postulated using a mathematical model of Ca^2+^ diffusion, referred to as the “Ca^2+^ microdomain” (Chad and Eckert, 1984; Fogelson and Zucker, 1985), and later experimentally observed in the squid giant presynaptic terminal (Llinás et al., 1992) and the frog neuromuscular junction (DiGregorio et al., 1999).

The term microdomain originally had a broad meaning, referring to the high concentration of Ca^2+^ near open VGCCs in general. More recently, the Ca^2+^ microdomain has also referred to a coupling between VGCCs and the vesicular Ca^2+^ sensor together with its counterpart Ca^2+^ nanodomain (Augustine et al., 2003; Fedchyshyn and Wang, 2005; Eggermann et al., 2012; Wang and Augustine, 2015; Bornschein and Schmidt, 2019). To date, there has been no direct measurement of the coupling distance between VGCCs and vesicular Ca^2+^ sensors by microscopic observation because of the technical limitation in identifying/labeling VGCCs together with synaptic vesicles. Alternatively, the quantitative estimate of the coupling distance via intraterminal loading of exogenous Ca^2+^ chelators. The widely used chelators EGTA and BAPTA [1,2-Bis(2-aminophenoxy)ethane-N,N,N′,N′-tetraacetic acid] have similar binding affinities, but the forward binding kinetics of BAPTA is ∼40 times faster than that of EGTA (Naraghi and Neher, 1997). The slow on rate of EGTA is thought to result in its inability to capture Ca^2+^ ions diffusing from VGCCs before they bind to vesicular sensors. By contrast, BAPTA can capture calcium ions, thereby inhibiting vesicular fusion. At locations distal to the VGCCs, both chelators should equally inhibit vesicular release. The magnitude of inhibition of transmitter release by these chelators can be a readout of the coupling distance. Thus, microdomain refers to the loose VGCC–sensor coupling distance that is inhibited by both EGTA and BAPTA, whereas nanodomain refers to the tight coupling inhibited solely by BAPTA (Augustine et al., 2003; Fedchyshyn and Wang, 2005; Wang and Augustine, 2015). The border separating the two domains is proposed to be 50–150 nm (Eggermann et al., 2012; Bornschein and Schmidt, 2019). This overall concept became widely recognized after the striking difference in the effectiveness of EGTA and BAPTA at the squid giant synapse was observed (Adler et al., 1991). The effects of these exogenous chelators have been further examined in a variety of nerve terminals (Borst et al., 1995; Rozov et al., 2001; Hefft and Jonas, 2005; Schmidt et al., 2013; Ritzau-Jost et al., 2014; Vyleta and Jonas, 2014).

However, EGTA might introduce some error when used as a diagnostic tool under certain conditions. Our recent studies using reaction diffusion simulations (RDSs) indicated that EGTA can inhibit vesicular release in the immediate vicinity (∼20 nm) of VGCCs, in the nanodomain coupling distance (Nakamura et al., 2015, 2018). Although an early study pointed out the ability of EGTA to reduce [Ca^2+^]_*i*_ at channel pores (Neher, 1986), little attention has been paid to this. In this article, I will examine how EGTA affects the Ca^2+^ microdomain around a single VGCC (single domain; Stanley, 2015) as well as the resultant vesicular release to precisely understand the inhibitory effect of EGTA on transmitter release.

## Materials and Methods

### Linearized Buffer Approximation (LBA)

First, I estimated local [Ca^2+^]_*i*_ around an open VGCC using linearized buffer approximation (LBA; Naraghi and Neher, 1997). Among several analytical solutions, LBA has been widely used to calculate local [Ca^2+^]_*i*_ because it reliably estimates [Ca^2+^]_*i*_ at an arbitrary distance from the channel in the presence of multiple Ca^2+^ buffer species (Neher, 1998a), without time-consuming numerical simulations. All parameters were the same as those in the RDS (see below), except that the endogenous fixed buffer (EFB) was inherently excluded from LBA (Naraghi and Neher, 1997). The calculations were performed using Microsoft Excel.

### Reaction Diffusion Simulations (RDS)

To estimate [Ca^2+^]_*i*_ gradients around an open VGCC, I mostly used RDS. In contrast to LBA, which calculates a single [Ca^2+^]_*i*_ at steady state (Naraghi and Neher, 1997), RDS can deduce time-dependent [Ca^2+^]_*i*_ changes in the presence and absence of buffers. Moreover, it can incorporate the geometry of the presynaptic terminal, which is of huge importance when analyzing diffusion in confined environments such as small presynaptic terminals. In this study, the same simulation environment and parameters were used as those described by Nakamura et al. (2018). Ca^2+^ entry, diffusion, and binding with buffers were calculated by numerically integrating partial differential equations applying an explicit finite-element (Euler) method using the Java-based simulation environment D3D. The total simulation volume was set to 1.0 (*x*) × 1.0 (*y*) × 1.0 (*z*) μm, which was divided into the elemental simulation voxels of 10 × 10 × 10 nm for finite difference calculations. With this voxel size, a concentration of 1 mM is equal to 602 molecules per voxel. The time step of simulation was 0.06 μs, and the resultant Ca^2+^ wave outputs were at 100 kHz. In this dimension, one simulation trial (10 ms in duration) typically took ∼7 h using a Windows 10 PC equipped with Intel Xeon CPU (E5-2640, 2.6 GHz).

A single VGCC was placed at the center of a single surface (*z* = 0). The current amplitude of a single VGCC was 0.3 pA, assuming the single channel conductance for Ca_*V*_2 is 2.7 pS (Weber et al., 2010) and the driving force for Ca^2+^ is ∼110 mV, when the terminal membrane potential at rest is −65 mV, and the reversal potential for Ca^2+^ is +43 mV in 2 mM extracellular Ca^2+^ (Sheng et al., 2012). Although the single VGCC current under physiological conditions can vary depending on membrane potential, the amplitude was fixed at 0.3 pA in this study, as our previous study using amplitudes ranging from 0.075 to 0.6 pA produced similar tendencies [see Figures 2, 4 in the study by Nakamura et al. (2018)]. This current amplitude corresponds to an influx of 936 Ca^2+^ ions per millisecond. The resting free [Ca^2+^] was set at 50 nM and the Ca^2+^ diffusion coefficient (*D*_*Ca*_) was 220 μm^2^/s (Allbritton et al., 1992). The parameters for buffers were set to match those of previous recordings performed in the dialyzed calyx of Held presynaptic terminal at room temperature (22–24°C) when possible (Nakamura et al., 2015). Free ATP was set at 200 μM, assuming the presence of 2 mM ATP (total concentration) and 3 mM Mg^2+^ (calculation using Maxchelator). The values for Ca^2+^ binding to ATP were as follows: *K*_*D*__,Ca_ = 200 μM, *k*_*on,Ca*_ = 5 × 10^8^ M^–1^s^–1^ (Baylor and Hollingworth, 1998), *D*_*ATP*_ = 220 μm^2^/s. The parameters for EGTA were *k*_*on*_ = 1.05 × 10^7^ M^–1^s^–1^ and *K*_*D*_ = 70 nM (Nägerl et al., 2000). The parameters for BAPTA were *k*_*on*_ = 4.0 × 10^8^ M^–1^s^–1^ and *K*_*D*_ = 220 nM (Naraghi and Neher, 1997). The diffusion coefficients of EGTA and BAPTA were 220 μm^2^/s (Naraghi and Neher, 1997). In the simulations with EFB, EFB was evenly distributed throughout the simulation volume. EFB and mobile buffers are similar, except that the EFB diffusion coefficient is 0 μm^2^/s. The parameters for EFB were as follows: *K*_*D*_ = 100 μM (Xu et al., 1997), *k*_*on*_ = 1 × 10^8^ M^–1^s^–1^, Ca^2+^ binding ratio (κ) = 40 (Helmchen et al., 1997). To focus on diffusion and buffering, Ca^2+^ extrusion was not implemented in this study. The resultant [Ca^2+^]_*i*_ waves were imported into IgorPro 8 (WaveMetrics) and analyzed using NeuroMatic (Rothman and Silver, 2018).

### Simulations of Vesicular Release Probability (Pv)

A five-site model of Ca^2+^-dependent vesicle fusion derived from experiments in the mature calyx of Held (Kochubey et al., 2009) was used to simulate vesicular transmitter release. The model was integrated using a forward Euler numerical integration routine (IgorPro 8), using the [Ca^2+^](*t*) generated from RDS. The probability over time of arriving in the fused state is considered the probability that a single vesicle will be released as a function of time (single vesicular release rate). The cumulative release rate, which is equivalent to the vesicular release probability (Pv), was estimated from the integral of the single vesicular release rate over a window from the onset of VGCC opening to 10 ms after closing. With the assumption that the Ca^2+^ sensors for vesicular release are juxtaposed to the release face membrane, Pvs were calculated only in the voxels on the synaptic surface (*z* = 0).

## Results

### Calculation of [Ca^2+^]_*i*_ Using Linearized Buffer Approximation

**FIGURE 1:**
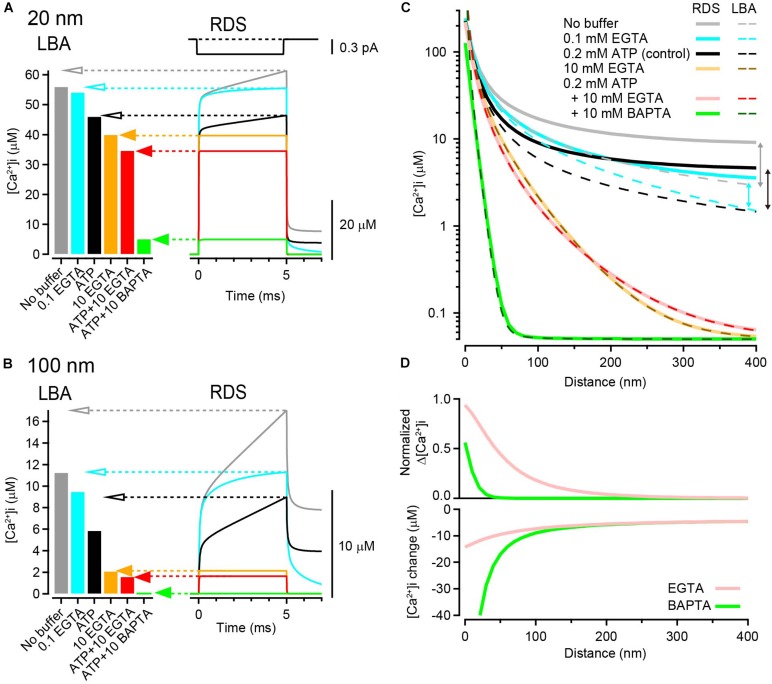
Calculation of [Ca^2+^]_*i*_ gradients around an open VGCC. **(A)** left, [Ca^2+^]_*i*_ at 20 nm from the VGCC calculated using LBA under various buffer conditions. Single channel current amplitude was 0.3 pA. Right, Time dependent [Ca^2+^]_*i*_ changes calculated using RDS. Channel open duration was 5 ms (top). Under heavily buffered conditions, both estimates agreed (filled arrows), whereas under the weak or no-buffer conditions, LBA predicted lower [Ca^2+^]_*i*_ than the peak [Ca^2+^]_*i*_ of transients in RDS (open arrows). Resting [Ca^2+^]_*i*_ was 50 nM. **(B)** Same as **(A)** but at 100 nm from the VGCC. **(C)** Spatial gradients of [Ca^2+^]_*i*_ from LBA (dashed) and RDS (solid) in various buffer conditions. The vertical arrows indicate discrepancies between the two estimates. **(D)** Reduction of [Ca^2+^] by 10 mM EGTA (pink) and BAPTA (green) against distance. Top, the reduction is represented as the ratio of the peak [Ca^2+^]_*i*_ in the presence of the chelator to that in the control (0.2 mM ATP). Bottom, the reduction is represented as the difference between the peak [Ca^2+^]_*i*_ in the presence of the chelator and that in the control.

To quantify the [Ca^2+^]_*i*_ gradient around a single open VGCC, I first used the analytical solution. When a calcium ion enters a channel pore, it is unbound (free ion) and starts diffusing. The increment in [Ca^2+^]_*i*_ (Δ[Ca^2+^]_*i*_) by diffusion is inversely proportional to the distance (*r*) from the channel pore. Assuming that Ca^2+^ is diffusing into a hemisphere adjacent to the plasma membrane, it is calculated by:

(1) $$\text{Δ}\,{\left[{\text{Ca}}^{2+}\right]}_{i}=i_{C\,a}/\left(4\,\pi \cdot F\cdot D_{C\,a}\cdot r\right)$$

where *i*_*Ca*_ is the amplitude of the constant Ca^2+^ current of a single VGCC, *F* is the Faraday constant, and *D*_*Ca*_ is the diffusion coefficient of free Ca^2+^ (Neher, 1986). When the single channel current amplitude was 0.3 pA (Weber et al., 2010), Δ[Ca^2+^]_*i*_ was 56.3 μM at 20 nm and 11.3 μM at a 100 nm distance in the absence of buffers (Figures 1A,B, left gray bars).

However, because several Ca^2+^ buffers are present in the presynaptic terminal, a calcium ion often binds to a buffer molecule before it reaches vesicular Ca^2+^ sensors; Ca^2+^ buffers dampen the Δ[Ca^2+^]_*i*_. To calculate the [Ca^2+^]_*i*_ gradient in the presence of buffers, I used LBA, which reliably estimates [Ca^2+^]_*i*_ in the immediate vicinity of VGCC in the presence of multiple mobile buffer species (Neher, 1998a). The theoretical background of this approximation is based on the assumption that a steady [Ca^2+^]_*i*_ gradient is immediately established upon an opening of VGCCs if the concentration of free Ca^2+^ buffer is very high compared with [Ca^2+^]_*i*_.

In the presence of an excess amount of buffer, it is assumed that Ca^2+^ influx through a VGCC does not substantially change the free buffer concentration. Under such conditions, a free calcium ion diffuses randomly until it is captured by a buffer molecule. This requires on the average time:

(2) $$\tau =1/\left(\left[B\right]\cdot k_{o\,n}\right)$$

where [*B*] and *k*_*on*_ represent the concentration and forward binding constant of the buffer, respectively. During this time, a calcium ion will travel a distance:

(3) $$\lambda =\surd (D_{C\,a}/\left(\left[B\right]\cdot k_{o\,n}\right)$$

The buffer decreases the average distance that calcium ions travel, and space constant λ is supplemented to Eq. 1. Thus, in the presence of buffer, Δ[Ca^2+^]_*i*_ at a given location is calculated by (for more detail, see Stern, 1992; Neher, 1998a; Ait-Haddou et al., 2010):

(4) $$\text{Δ}\,{\left[C\,a^{2+}\right]}_{i}=i_{C\,a}/\left(4\,\pi \cdot F\cdot D_{C\,a}\cdot r\right)\cdot e\,x\,p\,\left(-r/\text{λ}\right)$$

λ may also be considered the border of the “non-equilibrium” domain (Neher, 1998b). If *r* >> λ, the contribution of diffusional Ca^2+^ to total [Ca^2+^]_*i*_ becomes minor as Δ[Ca^2+^]_*i*_ becomes close to zero. In this distance range, the buffering reaction is at an equilibrium. By contrast, if *r* << λ, the value calculated for Eq. 4 becomes close to that for Eq. 1, indicating that diffusion primarily determines [Ca^2+^]_*i*_. Ca^2+^ exists as free ions and is not at an equilibrium. Within this non-equilibrium domain, because free buffer molecules and Ca^2+^-bound buffer molecules diffuse each other, the Ca^2+^ concentration remains at a steady state (Neher, 1998a). Space constant λ can be used to indicate the extent of the Ca^2+^ microdomain determined by the buffer.

According to Eqs 2 and 3, τ and λ for 0.1 mM EGTA were 1.63 ms and 599 nm, respectively. Thus, when the basal resting [Ca^2+^]_*i*_ was 50 nM, LBA calculates [Ca^2+^]_*i*_ as 54.4 μM at 20 nm and 9.6 μM at 100 nm, slightly decreased from the no-buffer condition (Figures 1A,B, left bar graphs). Similarly, [Ca^2+^]_*i*_ in the presence of 10 mM EGTA (τ = 16 μs, λ = 59.9 nm) was 40.3 μM at 20 nm and 2.2 μM at 100 nm. The addition of free ATP (0.2 mM), which widely exists in the cytosol and acts as a rapid-binding low-affinity mobile buffer, further decreased [Ca^2+^]_*i*_, and 10 mM BAPTA (τ = 0.3 μs, λ = 8.2 nm) strongly decreased [Ca^2+^]_*i*_ to 4.8 μM even at a distance of 20 nm (in the presence of ATP).

### Calculation of [Ca^2+^]_*i*_ Using Reaction Diffusion Simulation

Although LBA is a useful method for calculating local [Ca^2+^]_*i*_ at an arbitrary distance from the VGCC, it has several limitations because of its assumptions. First, under low-buffered conditions, LBA does not provide reliable estimates because Ca^2+^ influx could substantially alter the free buffer concentration. Second, as LBA assumes an instantaneous establishment of steady-state Δ[Ca^2+^]_*i*_ gradient after channel opening and calculates the steady-state Δ[Ca^2+^]_*i*_, it does not provide for time-dependent changes in [Ca^2+^]_*i*_. To circumvent these limitations, I next estimated local [Ca^2+^]_*i*_ gradients using RDS. To simulate time-dependent [Ca^2+^]_*i*_ changes, I used a single channel current of 0.3 pA (Weber et al., 2010) for 5 ms. The resultant [Ca^2+^]_*i*_ transient at 20 nm rose immediately after channel opening and gradually increased further until the end of the Ca^2+^ current. The peak [Ca^2+^]_*i*_ was 62 μM in the absence of Ca^2+^ buffers (Figure 1A right, gray trace). The addition of free ATP (0.2 mM) reduced the peak [Ca^2+^]_*i*_ to 47 μM by affecting both the fast and slow components (Figure 1A, black). By contrast, the addition of a low concentration (0.1 mM) of EGTA reduced the peak [Ca^2+^]_*i*_ to 56 μM, only affecting the slow component (Figure 1A, blue). Increasing the EGTA concentration to 10 mM eliminated this slow component and resulted in a steady-state [Ca^2+^]_*i*_ at 40 μM (Figure 1A, orange and red). Steady-state [Ca^2+^]_*i*_ was also attained in the presence of 10 mM BAPTA but with an amplitude as small as 5 μM (Figure 1A, green). At a 100 nm distance, [Ca^2+^]_*i*_ transients became smaller in amplitude with slower rising kinetics (Figure 1B).

When comparing [Ca^2+^]_*i*_ from LBA with the peak [Ca^2+^]_*i*_ from RDS, the two estimates matched in the presence of 10 mM EGTA or BAPTA (Figures 1A,B, filled arrows). Indeed, [Ca^2+^]_*i*_ immediately reached steady state in RDS under these highly buffered conditions. By contrast, LBA deduced smaller [Ca^2+^]_*i*_ under weak- or no-buffer conditions than RDS (open arrows). This difference became apparent in the plot showing entire spatial [Ca^2+^]_*i*_ gradients (Figure 1C). The discrepancy is partly accounted by the difference in diffusional spaces (Nakamura et al., 2018). Many analytical solutions, including LBA, assume an infinite diffusion volume, whereas RDS uses limited diffusional space, which tends to cause an accumulation of Ca^2+^. Furthermore, under weakly buffered conditions, Ca^2+^ influx substantially alters the free buffer concentration, violating the assumption of a linear buffer regime for LBA. The slower component of [Ca^2+^]_*i*_ in RDS is due to the limited diffusional space and the lower buffer condition. Thus, to estimate of [Ca^2+^]_*i*_ gradient in low-buffered condition, the use of RDS is preferable. However in highly buffered condition, LBA provides a powerful method for estimating steady-state [Ca^2+^]_*i*_, as there is a good agreement between the two estimates throughout the entire spatial gradients (Figure 1C).

To quantify the inhibition of the spatial gradient of [Ca^2+^]_*i*_ by EGTA and BAPTA, I compared Δ[Ca^2+^]_*i*_ under the control condition (0.2 mM ATP) with that in the presence of 10 mM chelators. As LBA cannot reliably predict [Ca^2+^]_*i*_ under low-buffered control conditions, I used [Ca^2+^]_*i*_ calculated from RDS. As predicted, both EGTA and BAPTA nearly completely inhibited Δ[Ca^2+^]_*i*_ at more distal locations (400 nm, <1% of control). By contrast, at a 10 nm distance from the VGCC, EGTA inhibited Δ[Ca^2+^]_*i*_ by only 15%, whereas BAPTA inhibited it by 83%. Thus, EGTA was much less potent in inhibiting Δ[Ca^2+^]_*i*_ than BAPTA at locations close to the channel (Figure 1D, top). However, it should be noted that EGTA still decreased [Ca^2+^]_*i*_ at this distance. When calculating the difference in [Ca^2+^]_*i*_ between control and chelator conditions, EGTA decreased the peak [Ca^2+^]_*i*_ by 13 μM, and BAPTA decreased [Ca^2+^]_*i*_ by 64 μM at a 10 nm distance (Figure 1D, bottom). Although EGTA was less potent than BAPTA, this reduction by EGTA was not negligible. These results indicate that a high concentration of EGTA can reduce [Ca^2+^]_*i*_ at all distances on the release surface, including the nanodomain of VGCCs.

### Buffer Property Affects the Rising Kinetics of the [Ca^2+^]_*i*_ Transient

**FIGURE 2:**
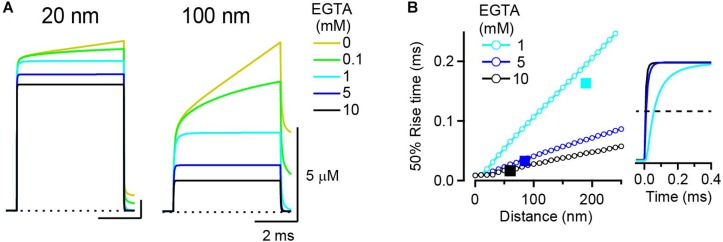
Rise kinetics of [Ca^2+^]_*i*_ transients. **(A)** [Ca^2+^]_*i*_ transient at 20 nm (left) and 100 nm (right) from a VGCC in the presence of various concentrations of EGTA. Single VGCC current was 0.3 pA in amplitude and 5 ms in duration. **(B)** Relationship between [Ca^2+^]_*i*_ transient kinetics and the distance; 50% rise time of [Ca^2+^] transients from RDS in the presence of 1, 5, and 10 mM EGTA was plotted against voxel distance from the VGCC. For comparison, the time constant [*τ* (ms)] from LBA plotted against the space constant [λ (nm)] for each EGTA concentration is denoted as a filled square. Traces in the right panel represent [Ca^2+^]_*i*_ transients (normalized with amplitude) at the voxel closest to the distance λ (190 nm for 1 mM; 80 nm for 5 mM; 60 nm for 10 mM EGTA). Horizontal dashed line denotes 50% rise of the transients.

I next examined the effect of buffers on the kinetics of the [Ca^2+^]_*i*_ transient. In the analytical solution, the average time to reach steady-state [Ca^2+^]_*i*_ after the onset of channel opening is inversely related to the forward binding kinetics and the concentration of buffers (see Eq. 2). When the EGTA concentration is, for example, 10 mM, τ is 16 μs (this is quite fast and approaches the assumption of LBA). However, because each free calcium ion randomly moves in a Brownian motion, the establishment of steady-state [Ca^2+^]_*i*_ might take longer at more distal locations. To examine this possibility, the 50% rise time of [Ca^2+^]_*i*_ transients was measured. At EGTA concentrations of <0.1 mM, [Ca^2+^]_*i*_ transients never reached steady state during the 5 ms channel opening, both at 20 nm and at 100 nm distances (Figure 2A). To attain steady-state [Ca^2+^]_*i*_ EGTA concentration of >1 mM was necessary. The 50% rise times at 20 nm were 19, 9.9, and 9.5 μs for 1, 5, and 10 mM EGTA, respectively. At 100 nm, they became longer, at 106, 41, and 28 μs, respectively. The rise time proportionally increased with the voxel distance (Figure 2B), indicating that steady-state [Ca^2+^]_*i*_ is attained in the proximity of the channel and then spreads to distal locations.

To compare these simulated rise times with the prediction from the analytical solution, I plotted τ (Eq. 2) against λ (Eq. 3) on the same graph (Figure 2B, squares). τ values for 5 and 10 mM EGTA appeared just on the lines that show a 50% rise time of RDS [Ca^2+^]_*i*_ transients against voxel distance, indicating that the ratio τ/λ corresponds to the slope of the 50% rise time of [Ca^2+^]_*i*_ transients against distance. However, τ for 1 mM EGTA went below its corresponding line. This was probably because the [Ca^2+^]_*i*_ transients in the RDS did not attain a true steady state. Indeed, in the presence of 1 mM EGTA, the peak amplitude of the [Ca^2+^]_*i*_ transient in RDS (Figure 2A, light blue trace) was slightly larger than the estimate from LBA (not shown); thus the 50% rise time was longer than τ. This means that EGTA concentrations of <1 mM deviate from a linear range and that the LBA should be avoided under these conditions.

### Effect of Endogenous Fixed Buffer on the [Ca^2+^]_*i*_ Transient

**FIGURE 3:**
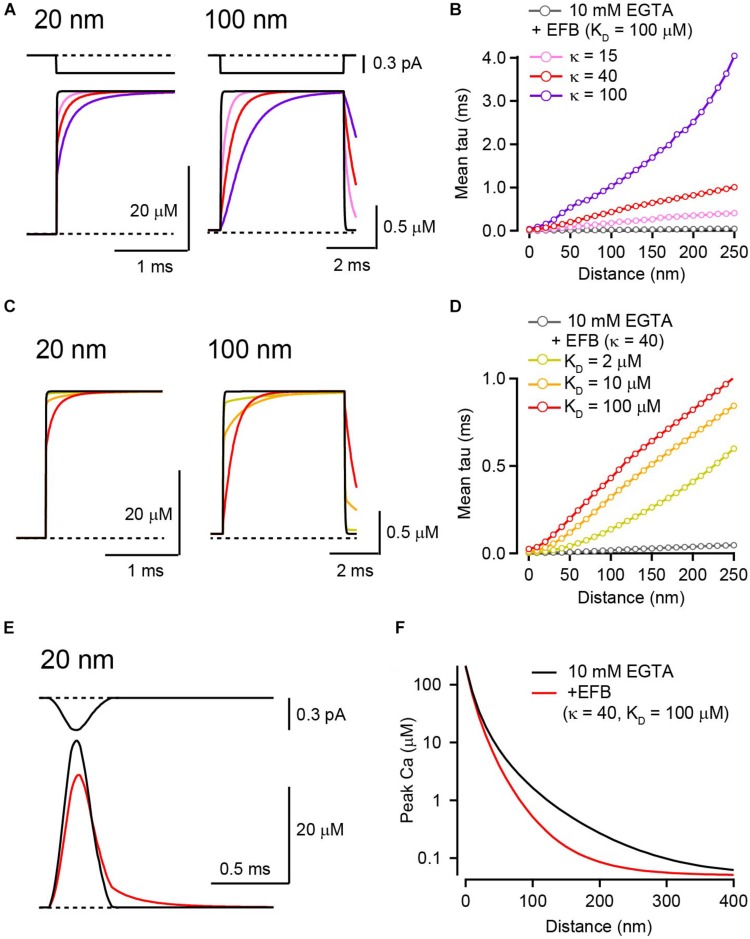
The effect of EFB on [Ca^2+^] transients in the presence of 10 mM EGTA. **(A)** Ca^2+^ current through a VGCC (top) and the resultant [Ca^2+^]_*i*_ transients for 20 nm (left) and 100 nm (right) distances in the presence of EFB with various κ values. I tested κ of 15 (pink), 40 (red), and 100 (purple) by altering EFB concentration. *K*_*D*_ was 100 μM. **(B)** Mean rise times of [Ca^2+^] transients for different EFB κ values were plotted against voxel distance. **(C)** [Ca^2+^] transients for 20 nm (left) and 100 nm (right) distances with various affinities of EFB. We tested *K*_*D*_ of 2 (yellow), 10 (orange), and 100 (red) μM. κ was set at 40. Black and red traces are identical to those in **(A)**. **(D)** Mean rise times of [Ca^2+^] transients for different EFB affinities. **(E)** Action potential-evoked [Ca^2+^] transients 20 nm from VGCC in the absence (black) and presence (red) of EFB. The current waveform (top) was obtained from whole-presynaptic terminal Ca^2+^ current at the calyx of Held evoked by action potential waveform voltage command and scaled to 0.3 pA. **(F)** Spatial gradients of peak [Ca^2+^] transients evoked by an action potential in the presence of low-affinity EFB.

In addition to mobile buffers, presynaptic terminals also contain an EFB (Helmchen et al., 1997; Neher and Taschenberger, 2013; Delvendahl et al., 2015; Nakamura et al., 2015). The exact molecular identity of the EFB is not known, but it is believed that ubiquitous proteins and lipids anchored to the membrane or cytoskeleton act as fixed low-affinity buffers (Schwaller, 2010). Calmodulin attached to presynaptic proteins (Eggermann et al., 2012) and Ca^2+^ sensors on vesicles might also act as EFBs. Calcium ions bound by mobile buffers retain their mobility because they diffuse together with their shuttle, whereas those bound by an EFB cannot diffuse. The EFB retards the distribution of calcium ions and delays the establishment of steady-state [Ca^2+^]_*i*_ by competing with mobile buffers. In LBA, the contribution of EFB is omitted because once the [Ca^2+^]_*i*_ reaches steady state, the standing [Ca^2+^]_*i*_ gradients are identical to those without an EFB (Stern, 1992; Naraghi and Neher, 1997). However, how long EFB might delay the establishment of steady-state [Ca^2+^]_*i*_ has not been examined in detail. To quantify the effect of EFB on the rise time of the [Ca^2+^]_*i*_ transient, I next tested EFBs with three different Ca^2+^ binding ratios (κ) and affinities. Because EFB κ is low in most presynaptic terminals, I used a κ of 15 (Neher and Taschenberger, 2013; Delvendahl et al., 2015) or 40 (Helmchen et al., 1997) but also tested a higher value (κ = 100). In the presence of 10 mM EGTA, the addition of low-affinity EFB (*K*_*D*_ = 100 μM) increased the time to reach steady state without affecting the steady-state amplitude of [Ca^2+^]_*i*_ (Figure 3A). The rising kinetics of the increase in [Ca^2+^]_*i*_ in the presence of EFB had multiple components, a rapid rise immediately after the onset of the Ca^2+^ current, followed by a gradual increase. Because the 50% rise time was not a reliable measure in this case, I then estimated the mean time constant to reach steady state. Increasing κ from 0 (10 mM EGTA alone) to 100 (10 mM EFB) increased the time constant for the [Ca^2+^]_*i*_ transient at all distances (Figure 3B). I next tested different *K*_*D*_s, including 2 (Meinrenken et al., 2002; Bucurenciu et al., 2008), 10, and 100 μM (Xu et al., 1997; DiGregorio et al., 1999; Nakamura et al., 2015), by altering *k*_*off*_. To keep κ constant (40), the EFB concentration was concomitantly adjusted in this simulation. The lowest *K*_*D*_ (100 μM) slowed the rise kinetics the most. At the 20 nm distance, the rise time increased ∼20-fold from 3.4 to 66 μs (Figures 3C,D).

In these simulations, EFB did not affect the amplitude of steady-state [Ca^2+^]_*i*_ for a 5 ms channel opening (Figures 3A,C). However, the slower rise kinetics of [Ca^2+^]_*i*_ transients in the presence of EFB might reduce the peak amplitude [Ca^2+^]_*i*_ evoked by brief channel openings. To test this possibility, a Ca current waveform evoked by the action potential in the mature calyx of Held (half duration, 0.19 ms; Nakamura et al., 2015) was used in the simulation, because this waveform is an example of the shortest evoked Ca^2+^ influx in the CNS and contrasts the longer square pulses. The addition of low-affinity EFB (*K*_*D*_ = 100 μM) reduced the peak [Ca^2+^]_*i*_ from 41 μM to 34 μM at 20 nm from VGCC (Figure 3E). The reduction in [Ca^2+^]_*i*_ was pronounced in more distal locations (Figure 3F) because Ca^2+^ influx terminated before the [Ca^2+^]_*i*_ reached steady state. Although I have presented the simulation result of EFB in the presence of 10 mM EGTA here, EFB also reduces the peak [Ca^2+^]_*i*_ in low concentrations of mobile Ca^2+^ buffers (see Nakamura et al., 2018). Thus, to estimate the [Ca^2+^]_*i*_ evoked by a brief Ca^2+^ influx, such as that induced by an action potential, the presence of EFB cannot be neglected and the RDS is preferred.

### Saturation of the Vesicular Release Sensor Masks the Reduction in [Ca^2+^]_*i*_

**FIGURE 4:**
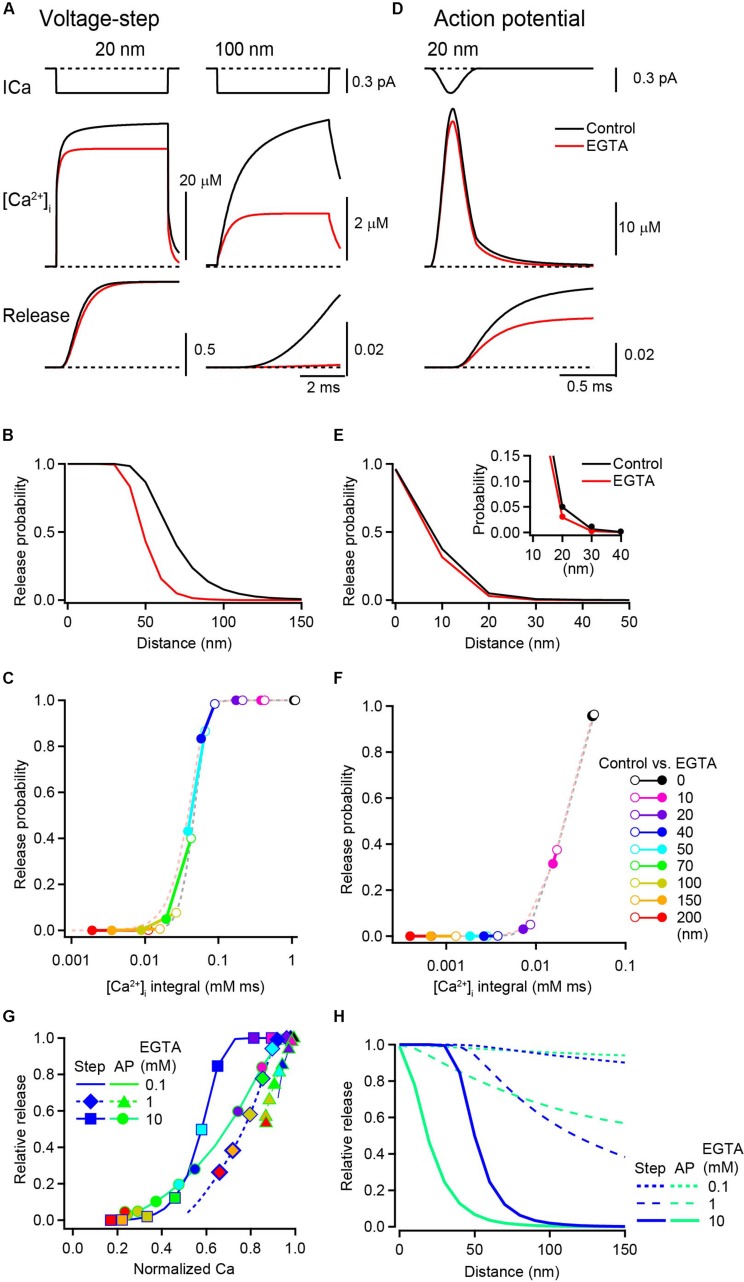
Inhibitory effect of EGTA on vesicular release probability. **(A)** [Ca^2+^] transients (middle) and the cumulative release probabilities (bottom) at 20 nm (left) and 100 nm (right) distances under the control condition (black) and in the presence of 10 mM EGTA (red), induced by a 5 ms depolarization. Single channel Ca^2+^ current is shown in the top row. **(B)** Spatial profile of Pv induced by voltage steps in the control (black) and in the presence of 10 mM EGTA (red). **(C)** Relationship between Pv and the time integral of [Ca^2+^]_*i*_ induced by voltage steps. Each circle pair represents Ca^2+^-Pv coupling under control (open) and EGTA (filled) conditions at various distances. Dashed lines represent the properties of Ca^2+^ secretion in relation to the vesicular sensor. **(D)** [Ca^2+^] transient (middle) and the resultant cumulative release probability (bottom) at 20 nm, evoked by an action potential. The top trace is a channel current in response to an action potential. **(E,F)** Same as in **(B,C)** but induced by action potentials. To better represent the decrease in Pv by EGTA at a 20 nm distance, the closer location is expanded in the inset. **(G)** The reduction in Pv by EGTA (Pv__*EGTA*_/Pv__*Control*_) was plotted against that in the time integral of [Ca^2+^]_*i*_ (Ca__*EGTA*_/Ca__*Control*_) at each location. Symbols on lines show the data from representative voxel distances from the VGCC [from left to right: 200, 150, 100, 70, 50, 40, 20, 10, and 0 nm, color code as in panel **(F)**]. 0.1 mM EGTA (dashed line) induced small reductions both in Ca^2+^ and Pv. Increased EGTA concentrations of 1 mM (diamonds and triangles on dashed lines) and 10 mM (circles and squares on solid lines) shifted the curve toward the lower left of the panel. **(H)** Spatial profiles of the inhibitory effect of various concentrations of EGTA on Pv evoked by voltage steps (blue) and by action potentials (green).

I next examined the effect of EGTA on the Pv, which was estimated from the waveform of [Ca^2+^]_*i*_ transients from RDS using the five-site model of Ca^2+^-dependent vesicular fusion (Kochubey et al., 2009). In this simulation, the control condition included 0.2 mM ATP and 4 mM low-affinity EFB. For the [Ca^2+^]_*i*_ transient induced by 5 ms voltage steps, the Pv at 20 nm rapidly (within 1 ms) reached 1 under the control condition. The addition of 10 mM EGTA reduced [Ca^2+^]_*i*_ and slightly slowed the rise of Pv, but the Pv similarly reached and remained at 1 (Figure 4A), indicating that EGTA had no effect on the final Pv at this distance. At 100 nm, an increase in Pv was observed only in the control condition, which was completely inhibited by EGTA. Under both control and EGTA conditions, the Pv decreased with distance, but this decrement occurred at a shorter distance in the presence of EGTA (Figure 4B). To quantify the relationship between Pv and Ca^2+^, Pvs were plotted against the time integral of [Ca^2+^]_*i*_ for each voxel location (Figure 4C). In proximity to VGCC (<40 nm in this example), EGTA caused a leftward shift of the points, indicating a decrease in [Ca^2+^]_*i*_. But because vesicular release sensors remained at a saturated level (*Pv* = 1), this decrease in [Ca^2+^]_*i*_ did not affect the Pv. At distal locations (>50 nm), the decrease in [Ca^2+^]_*i*_ by EGTA was seen as a decrease in the Pv, because the vesicular Ca^2+^ sensor entered a linear range.

Next, Pv with action potentials was examined. For this brief Ca^2+^ influx, EGTA reduced the peak [Ca^2+^]_*i*_ from 41 μM to 35 μM (by 15%) and the Pv from 0.07 to 0.03 (by 52%) at a 20 nm distance from the VGCC (Figure 4D). The Pv also decreased with distance, but this decrement occurred at shorter distance than for the voltage step (Figure 4E). For action potential-induced brief Ca^2+^ influx, the vesicular Ca^2+^ sensor showed no indication of saturation (Figure 4F). As the time integral of [Ca^2+^]_*i*_ at the channel pore (0 nm) was already in or near the linear range of the vesicular sensor, even a small decrease in [Ca^2+^]_*i*_ directly decreased the Pv. To examine how EGTA differently affects the Ca^2+^ release relationship for voltage steps and action potentials, the change in Pv (EGTA/control) was plotted against that in [Ca^2+^]_*i*_ in each voxel location. For voltage step-evoked release, the decrease in the Pv occurred when 10 mM EGTA reduced the [Ca^2+^]_*i*_ to 60% or less (Figure 4G, squares on solid blue line). For action potential-evoked release, the smaller decrease in the [Ca^2+^]_*i*_ (to ∼80%) decreased the Pv (circles on solid green line). For lower EGTA concentrations, the decreases in Pv and [Ca^2+^]_*i*_ were smaller (triangles and diamonds on dashed lines).

The results from the simulation and analysis indicate that the saturation of vesicular sensors primarily determines the inhibition of Pv by EGTA. In the proximity of VGCCs, voltage step-induced large [Ca^2+^]_*i*_ transients saturate vesicular sensors masking the effect of EGTA on release. By contrast, action potential-induced brief Ca^2+^ influx drives the Pv within a linear range of the sensor, revealing the effect of EGTA on the inhibition of vesicular release. At the same distance, the magnitude of inhibition of release by EGTA varies depending on the duration of Ca^2+^ influx (Figure 4H). In addition to the two above-described Ca^2+^ influx waveforms, I performed simulations using a Ca^2+^ current step followed by tail Ca^2+^ currents (Supplementary Figure S1). The spatial profile of the effects of EGTA on release also shifted depending on the duration of the Ca^2+^ current step. Thus, the duration of Ca^2+^ influx is a key determinant of the magnitude by which EGTA inhibits vesicular release (Nakamura et al., 2018).

### Duration of Ca^2+^ Influx Alters the Spatial Profile of the EGTA Effect on Transmitter Release

**FIGURE 5:**
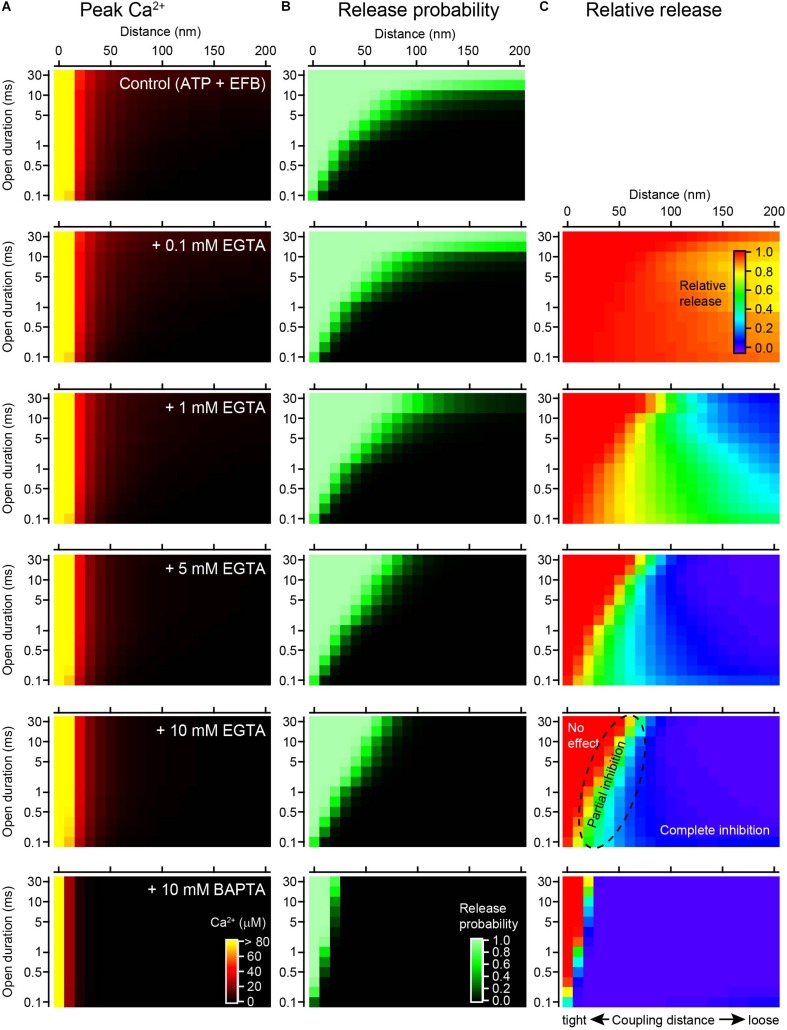
Channel open duration affects the inhibitory effect of EGTA on vesicular release. **(A)** Spatiotemporal profiles of peak [Ca^2+^]_*i*_ in response to a single channel opening under the control condition (0.2 mM ATP + EFB) and with 0.1, 1, 5, and 10 mM EGTA and 10 mM BAPTA (from top to bottom). The amplitude of the single VGCC current was 0.3 pA. Note that [Ca^2+^]_*i*_ of >80 μM is colored yellow to magnify the concentration gradient near *K*_*D*_ of the Ca^2+^ sensor. **(B)** Spatiotemporal profiles of Pvs calculated from Ca^2+^ transients in panel **(A)** using the five-site release model. **(C)** Spatiotemporal profiles of the inhibitory effect of various chelator concentrations of EGTA and 10 mM BAPTA on Pv. The graph for each chelator condition was calculated from the ratio of Pv in the chelator to that in the control.

To more systematically examine how the duration of channel opening influences the effect of EGTA, the step duration was varied from 0.1 to 30 ms. In this simulation, [Ca^2+^]_*i*_ transients evoked by brief steps were regarded as equivalent to those evoked by action potentials, whereas [Ca^2+^]_*i*_ transients evoked by longer influxes corresponded to voltage step-induced VGCC currents. ATP (0.2 mM) and the low-affinity EFB (4 mM) were included in this simulation. The peak [Ca^2+^]_*i*_ produced a spatiotemporal gradient (Figure 5A), but the spatial gradient (horizontal) was more apparent than the temporal gradient (vertical), especially for higher concentrations (>1 mM) of EGTA. This is because the steady-state [Ca^2+^]_*i*_ rapidly established in the presence of the buffer diminished the temporal gradient (see Figure 1). The gradients for the Pv values (Figure 5B) were less steep than those for [Ca^2+^]_*i*_, because the sensor saturation masked the [Ca^2+^]_*i*_ change at higher concentrations. The sensor saturation (shown as light green) covered the upper left corners of the graphs, in the proximity of the VGCC, and expanded along the temporal axis. An increase in the EGTA concentration decreased the Pv, especially at distal locations for long channel openings. By contrast, at close locations, EGTA minimally affected Pv.

To visualize the inhibitory effect of EGTA on release, I calculated the ratios of Pv (various concentrations of EGTA/control, Figure 5C). In the presence of 0.1 mM EGTA, most of the area was red colored, representing no effect of EGTA. A reduction in release was observed only at distal locations for longer channel openings. An increase in the EGTA concentration enlarged the blue area, representing complete inhibition of release. In the presence of 10 mM EGTA, the blue occupied the area from the distal end to 50 nm from the VGCC. On the contrary, the red area was restricted to the proximity of the VGCC (<50 nm) for long durations of channel opening (>20 ms). For very brief channel openings (<0.5 ms), the red area was further restricted to within 10 nm. In the intermediate area shown in the color gradient, EGTA partially inhibited vesicular release. As predicted, 10 mM BAPTA further inhibited the release.

In summary, EGTA inhibits vesicular release at locations distal from the VGCC for a wide range of channel open durations. At distances greater than 100 nm, EGTA completely diminishes the Pv, regardless of how long the channel is open. By contrast, the effect of EGTA in the vicinity of the channel critically depends on the time the channel is open; EGTA does not affect the Pv when the channel is open for a long time but decreases the Pv for brief openings. Thus, the duration of Ca^2+^ influx critically determines the inhibitory effect of EGTA on neurotransmitter release.

## Discussion

To precisely understand the effect of EGTA on the spatiotemporal gradients of [Ca^2+^]_*i*_ and vesicular release, I performed RDS of [Ca^2+^]_*i*_ transients following a single VGCC opening and simulations of vesicular release using a simplified version of the presynaptic terminal. RDS indicated that EGTA decreases [Ca^2+^]_*i*_ in the proximity of the channel (Figure 1D). Although this decrement in the [Ca^2+^]_*i*_ accounted for a minor fraction of the total [Ca^2+^]_*i*_, the subsequent release simulation suggested that EGTA decreases release, especially for a brief Ca^2+^ influx, such as that evoked by action potentials (Figures 4E–G). Thus, EGTA can inhibit vesicular release, at least partially, in the nanodomain of VGCCs. The saturation of vesicular sensors near the VGCC accounts for the lack of an effect of EGTA on vesicular release. EGTA decreases [Ca^2+^]_*i*_ at all distances from the VGCC, but the [Ca^2+^]_*i*_ in the presence of EGTA is sufficient to saturate sensors close to the channel when the duration of Ca^2+^ influx is long. In such cases, one cannot observe the decrease in [Ca^2+^]_*i*_ as a reduction in release. I propose that the property of the vesicular sensor and the Ca^2+^ influx duration critically determine the inhibitory effect of EGTA on release. When interpreting the results from synaptic experiments showing an inhibition of vesicular transmitter release by EGTA, these biophysical parameters must be considered.

### The Estimation of Coupling Distance Using EGTA in Combination With RDS

For more than two decades, EGTA has been widely tested in a variety of synapses to probe the coupling distance between VGCCs and vesicular Ca^2+^ sensors. insights from analytical solutions including LBA (Naraghi and Neher, 1997), the lack of the EGTA effect on vesicular transmitter release has been explained by an the inaccessibility of EGTA to calcium ions in the proximity of the VGCC and is thus regarded as an indication of nanodomain coupling. On the contrary, the inhibition of vesicular release by EGTA is considered an indication of microdomain coupling (Augustine et al., 2003; Eggermann et al., 2012; Wang and Augustine, 2015; Bornschein and Schmidt, 2019). However, the discrimination of the nanodomain from the microdomain solely on the basis of the inhibitory effect of EGTA is less physiologically relevant, because the excessively buffered conditions achieved experimentally with exogenous chelators are artificial. In the unperturbed presynaptic terminal, the spatial extent of the Ca^2+^ microdomain can be determined by the diffusion coefficient, and by the product of the concentrations and binding kinetics of the endogenous buffers (Eq. 3). With the assumption that buffer properties vary among cell types, the absolute length λ is cell specific and can range from orders of nanometers to micrometers. Whether the vesicular Ca^2+^ sensors are located within or beyond this λ is more important because the effects of endogenous Ca^2+^ buffers on Pv and short-term plasticity depend on the distance of the sensor from the VGCCs. In this context, the physical distance between vesicular Ca^2+^ sensors and VGCCs (coupling distance) is important. EGTA should be used to measure the absolute distance rather than to discriminate nano/microdomains. The knowledge of the absolute distance would help to understand the complex molecular composition governing vesicular release.

Reaction diffusion simulation can provide valuable insights regarding the effect of EGTA on the spatial gradients of [Ca^2+^]_*i*_ around VGCCs. This simulation has several advantages, including the incorporation of the time course of Ca^2+^ entry and the property of endogenous buffers, including EFBs. Moreover, by combining release simulations, we can incorporate the property of the vesicular sensor to estimate the effect of EGTA on release. In this study, I focused on the Ca^2+^ microdomain formed around a single channel (single domain; Stanley, 2015). However, RDS can be used for the more complex geometry of presynaptic terminals, including for the distribution of VGCCs at the release site (Nakamura et al., 2015; Miki et al., 2018). As computer power has increased enormously, RDS has become less time consuming. EGTA and simulations together make a powerful diagnostic tool for estimating the coupling distance. Figure 5C provides useful insight into past and future experiments. In the following sections, I will review some of the previous synaptic experiments using EGTA and discuss how we can interpret them on the basis of the results from the present simulation study.

### No Inhibition

The lack of an effect of EGTA on neurotransmitter release has been reported for a variety of central synapses. Bath application of EGTA-AM has a negligible effect on the amplitude of excitatory postsynaptic currents (EPSCs) in climbing fiber-Purkinje synapses (Matsui and Jahr, 2003), granule cell-to-molecular layer interneuron synapses (Satake and Imoto, 2014), and mature cerebellar granule cell-to-Purkinje cell synapses (Schmidt et al., 2013; Baur et al., 2015). EGTA-AM also shows no effect on inhibitory postsynaptic currents (IPSCs) in synapses between molecular layer interneurons (Christie et al., 2011) or between hippocampal basket cells and granule cells (Hefft and Jonas, 2005). Unfortunately, as the cytosolic EGTA concentration is uncertain in these experiments using this membrane permeable derivative of EGTA, it is impossible to estimate the absolute coupling distance. However, because the duration of channel opening must be as brief as 0.5 ms for these action potential-evoked transmitter releases, the data are likely situated near the left bottom corners of the plots in Figure 5C. Thus, only a tight coupling between vesicular sensors and channels can account for the experimental observation that EGTA has no effect on release.

A lack of an effect with EGTA has also been reported for voltage step stimulation with longer channel openings. EGTA (5 mM) does not affect the fast release components for responses at cerebellar mossy fiber-granule synapses (Ritzau-Jost et al., 2014), Purkinje cell and granule cell terminals in culture (Kawaguchi and Sakaba, 2015, 2017), and the mature calyx of Held (Chen et al., 2015). A high concentration of EGTA also does not affect the fast release component of responses at auditory hair cell ribbon synapses (Moser and Beutner, 2000; Johnson et al., 2017) or retinal ribbon synapses (Singer and Diamond, 2003). These fast release components are thought to be mediated by vesicular sensors near the channels (Wadel et al., 2007; Chen et al., 2015) because the [Ca^2+^]_*i*_ at distal locations is not sufficient to trigger vesicular release under high-EGTA conditions (Figure 1C). In response to voltage step-induced long Ca^2+^ influx, Ca^2+^ sensors near the channels are likely to be saturated; thus EGTA effect cannot be seen as the reduction of release. The distance from the channel at which EGTA shows no inhibition can range from 0 to 50 nm depending on channel open duration.

The most famous example showing a lack of an effect of EGTA was at the squid giant synapse, in which ∼80 mM EGTA had no effect on transmitter release but BAPTA diminished it (Adler et al., 1991). Although this pioneer study opened the door to this field, the squid synapse is unique, such that a high concentration of EGTA had no effect on action potential-evoked, i.e., a brief Ca^2+^ influx-induced, vesicular release. In mammalian synapses, EGTA is typically tested at concentrations ranging between 1 and 30 mM, and the high concentration (>10 mM) resulted in some inhibition of release. There are several possible reasons for the difference between squid and mammalian synapses. First, the forward binding of EGTA is slower in the squid synapse because of the lower temperature and higher ionic concentration. Thus, it is possible that the [Ca^2+^]_*i*_ gradient in the presence of EGTA in squid synapses is less steep than that in mammalian terminals. Second, the time course of Ca^2+^ entry during a presynaptic action potential (∼1 ms; Llinás et al., 1981) is longer than that in typical mammalian synapses (Borst and Sakmann, 1998; Bischofberger et al., 2002; Ritzau-Jost et al., 2014; Nakamura et al., 2015), increasing the time window for the lack of an observed effect of EGTA. Third, the higher external [Ca^2+^] causes a larger single channel current, enhancing the saturation of sensors. All these factors might contribute to the reduced effect of EGTA at the squid synapse. Although Adler et al. (1991) accounted for these factors and adjusted the EGTA parameters, these biophysical parameters in the squid should be investigated further.

### Complete Inhibition

Our simulations indicate that complete inhibition of release by EGTA is robust evidence for “microdomain” coupling. If the sensor is located beyond 100 nm from a channel, EGTA completely inhibits vesicular release regardless of how long the channel is open (Figure 5C). However, complete inhibition of action potential-induced vesicular release by EGTA has not been reported, at least for the first pulse of evoked synchronous release. This is partly because an action potential-evoked brief Ca^2+^ influx thorough single VGCC hardly induce detectable Pv at distal locations from the channel (Figure 5B; Stanley, 2015). For synchronous release, EGTA most often exhibits no effect or partial inhibition. Complete inhibition by EGTA was demonstrated for delayed release at cerebellar granule cell-to-stellate cell synapses (Atluri and Regehr, 1998), and asynchronous release at autaptic hippocampal synapses (Otsu et al., 2004) and retinal ribbon synapses (Singer and Diamond, 2003). Although there is debate regarding the origins, pools, and sensors for these different release modes (Schneggenburger and Rosenmund, 2015), it is interesting to examine whether asynchronous release might occur at more distal locations relative to synchronous release. The 5-site release model (Schneggenburger and Neher, 2000; Kochubey et al., 2009) used in the present study encompasses the [Ca^2+^]_*i*_ range for synchronous release. To investigate the coupling distance for asynchronous, delayed, and spontaneous release, other Ca^2+^ sensor models incorporating low [Ca^2+^]_*i*_ (Lou et al., 2005) might be more useful. Nevertheless, the comparison of BAPTA and EGTA provides clues on the coupling distance for spontaneous release (Goswami et al., 2012; Williams et al., 2012).

### Partial Inhibition

At most central synapses, high concentrations of EGTA partially inhibit vesicular release. This experimental observation and its underlying mechanism should be interpreted with caution because the coupling distance generating partial inhibition ranges several tens of nanometers (Figure 5C), covering nanodomain to microdomain distances (depending on the definitions). In response to a long voltage step, vesicular release has fast and slow release components. Although the fast component is insensitive to EGTA, 5–20 mM EGTA reduces the slow component at cerebellar mossy fiber-granule synapses (Ritzau-Jost et al., 2014), the mature calyx of Held (Chen et al., 2015), and hair cell ribbon synapses (Moser and Beutner, 2000; Johnson et al., 2017). The slow components might use different Ca^2+^ sensors (Wölfel et al., 2007), but could be explained by release from locations distal from the channel (Wadel et al., 2007) because 20 mM EGTA preferentially blocks vesicular release at >50 nm (Chen et al., 2015). Thus, for experiments using long voltage steps, a high concentration of EGTA dissects the location of vesicular release and can be used as a diagnostic tool to discriminate nanodomain and microdomain coupling distances.

Partial inhibition by EGTA has also been reported for action potential-evoked release. The first example reported in a central synapse was for the calyx of Held, in which presynaptic loading with 10 mM EGTA from a patch pipette reduced action potential-evoked release (Borst and Sakmann, 1996). Although the effect of EGTA becomes smaller throughout postnatal development, EGTA inhibits EPSCs by 22–69% (Fedchyshyn and Wang, 2005; Nakamura et al., 2015). At synapses between layer 2/3 pyramidal neurons and interneurons, the EPSC is reduced by dialyzing millimolar concentrations of EGTA into the presynaptic pyramidal neuron (Rozov et al., 2001). In hippocampal autaptic synapses, EGTA-AM reduces EPSCs by 20% (Otsu et al., 2004). IPSCs at hippocampal basket cell-to-granule cell synapses are reduced by injecting the presynaptic cell with 30 mM EGTA (Bucurenciu et al., 2008). Similarly, 10–30 mM EGTA reduces IPSCs at cerebellar basket cell-Purkinje cell synapses (Arai and Jonas, 2014). The inhibitory effect of EGTA on postsynaptic currents was originally thought to indicate loose “microdomain” coupling, but more recent simulations indicate that the coupling distance ranges from 10 to 20 nm in these inhibitory terminals (Bucurenciu et al., 2008; Arai and Jonas, 2014) and from 10 to 40 nm in the calyx depending on the developmental stage (Nakamura et al., 2015). Direct patch clamp recordings from the calyx revealed that the half duration of Ca^2+^ influx during an action potential is 0.36 ms (Borst and Sakmann, 1998). This brief Ca^2+^ influx prevents the sensor from becoming saturated; thus the reduction in presynaptic [Ca^2+^]_*i*_ by EGTA immediately affects vesicular release. Indeed, it was shown that Ca^2+^ sensors are not saturated at these synapses because higher external [Ca^2+^] increases Pv (Bucurenciu et al., 2008; Koike-Tani et al., 2008). As with these studies, the possibility of sensor saturation should be always examined at the synapse. Presynaptic Ca^2+^ uncaging experiments can also provide information on vesicular Ca^2+^ sensors (Schneggenburger and Neher, 2000).

A robust inhibitory effect of EGTA was also reported for the hippocampal mossy fiber-CA3 pyramidal cell synapse, in which 10 mM EGTA reduces EPSCs to merely 8% (Vyleta and Jonas, 2014). Combined with the BAPTA experiments, the coupling distance is estimated at ∼70 nm. Whether this loose coupling can be classified as “microdomain” distance depends on the definition and on the number of Ca^2+^ channels triggering release (Bornschein and Schmidt, 2019). As the duration of Ca^2+^ influx during an action potential is short (0.58 ms; Bischofberger et al., 2002), the coupling distance at this synapse can be determined via plots, as in Figure 5C.

## Conclusion

The present study sheds light on two points that have received little attention: the slow Ca^2+^ chelator EGTA can access Ca^2+^ ions to decrease [Ca^2+^]_*i*_ in the proximity of a channel, and the saturation of the vesicular sensor masks the effect of EGTA on vesicular release. When Ca^2+^ influx is triggered by an action potential, a small reduction of [Ca^2+^]_*i*_ by EGTA causes an immediate reduction of vesicular release because the vesicular Ca^2+^ sensor is in a linear range. Thus, the experimental observation that EGTA inhibits synaptic transmission alone does not directly indicate that the coupling is within the microdomain. A more accurate interpretation requires the consideration of additional biophysical parameters, of which, the most important is the duration of Ca^2+^ influx.

## Data Availability Statement

All datasets generated for this study are included in the manuscript/Supplementary Files.

## Author Contributions

YN conceived the research, performed the simulation, wrote the manuscript, and prepared the figures.

## Conflict of Interest

The author declares that the research was conducted in the absence of any commercial or financial relationships that could be construed as a potential conflict of interest.

## Supplementary Material

FIGURE S1 The effect of tail Ca^2+^ currents on [Ca^2+^]_*i*_ and release simulations. **(A)** Top, Ca^2+^ current waveforms used in this simulation consisted of steady step current (0.15 pA) and subsequent tail current. Durations of step currents varied from 0.5 to 5 ms. The peak amplitude of the tail current was 0.3 pA, which decayed exponentially with the fast (0.2 ms, 90%) and slow (1.0 ms, 10%) time constants. Middle, [Ca^2+^]_*i*_ transients at a 20 nm distance in the control (0.2 mM ATP and EFB, solid line) and in the presence of 10 mM EGTA (dashed line). The tail current additionally increased [Ca^2+^]_*i*_ upon the steady-state [Ca^2+^]_*i*_ induced by the preceding step current. Bottom, cumulative release probability. Pv increased with the duration of the step. **(B)** Spatial profiles of Pvs evoked using Ca^2+^ current with tail current in the control and in the presence of EGTA. **(C)** Spatial profiles of the inhibitory effect of EGTA on release. Spatial profile shifted with the increase in step duration. **(D–F)** Same as **(A–C)** but without tail current. When the duration of step component was sufficiently long (e.g., 5 ms, red traces), the spatial profiles of Pv **(B,E)** and the effect of EGTA on release **(C,F)** showed similar distributions, because Pv and the effect of EGTA were exclusively determined by the [Ca^2+^]_*i*_ increase during the step. By contrast, when the step duration was shorter (e.g., 0.5 ms, blue traces), the tail current additionally elevated Pv, and the effect of EGTA decreased. **(G)** For comparison, release rate traces under the control condition for 0.5 and 5 ms steps with/without tail current are shown. This simulation indicates that the tail current significantly contributes only when the channel is open for a short time. It should be noted that an action potential-induced brief Ca^2+^ current is mostly mediated by a tail component.
