# Supplementary figures and images for: EGTA Can Inhibit Vesicular Release in the Nanodomain of Single Ca2+ Channels
Source: Front Synaptic Neurosci. 2019 Oct 1;11:26. doi: 10.3389/fnsyn.2019.00026 (PMC6779814; doi:10.3389/fnsyn.2019.00026)

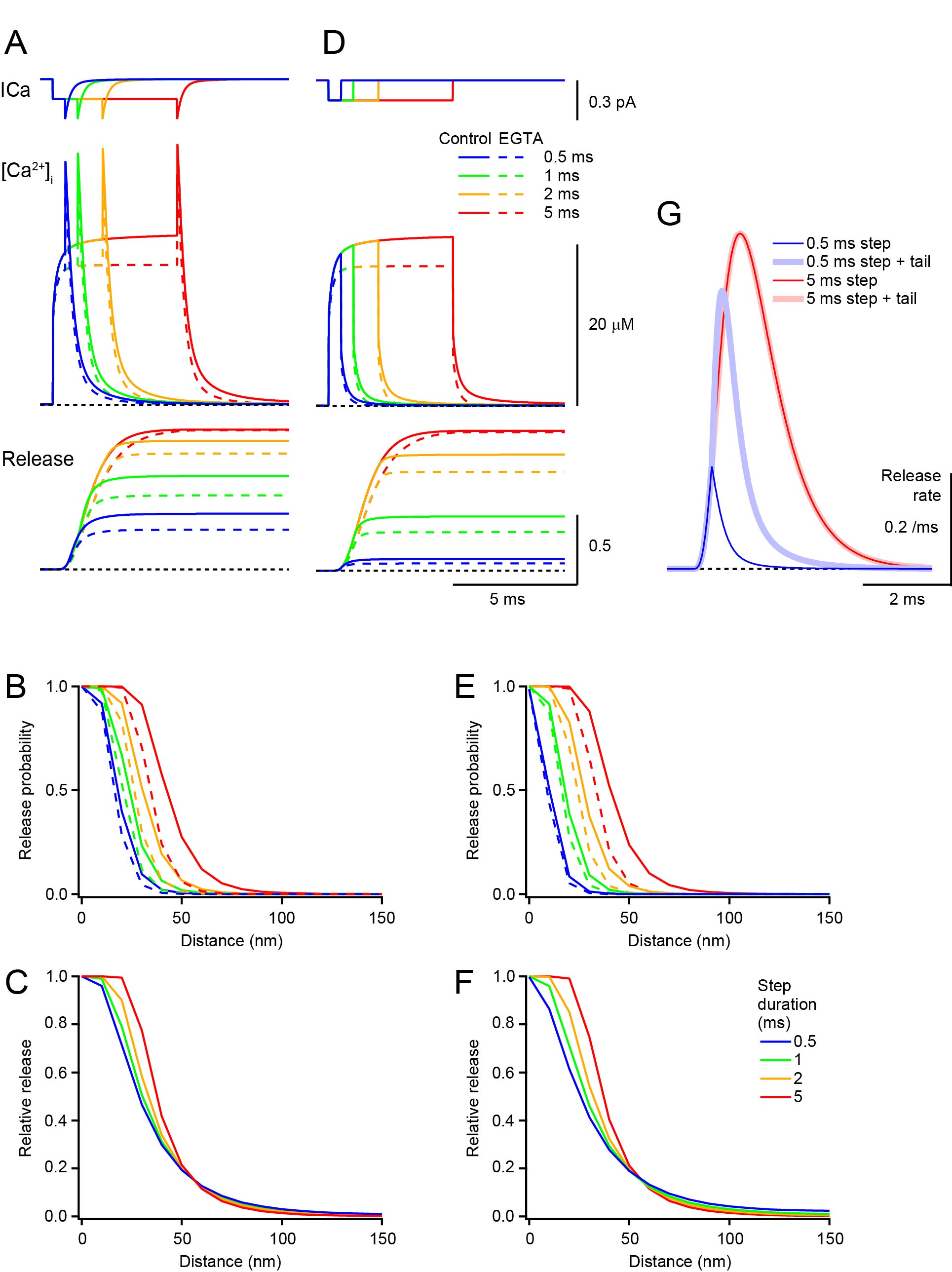

Supplement: FIGURE S1 — The effect of tail Ca2+ currents on [Ca2+]i and release simulations. (A) Top, Ca2+ current waveforms used in this simulation consisted of steady step current (0.15 pA) and subsequent tail current. Durations of step currents varied from 0.5 to 5 ms. The peak amplitude of the tail current was 0.3 pA, which decayed exponentially with the fast (0.2 ms, 90%) and slow (1.0 ms, 10%) time constants. Middle, [Ca2+]i transients at a 20 nm distance in the control (0.2 mM ATP and EFB, solid line) and in the presence of 10 mM EGTA (dashed line). The tail current additionally increased [Ca2+]i upon the steady-state [Ca2+]i induced by the preceding step current. Bottom, cumulative release probability. Pv increased with the duration of the step. (B) Spatial profiles of Pvs evoked using Ca2+ current with tail current in the control and in the presence of EGTA. (C) Spatial profiles of the inhibitory effect of EGTA on release. Spatial profile shifted with the increase in step duration. (D–F) Same as (A–C) but without tail current. When the duration of step component was sufficiently long (e.g., 5 ms, red traces), the spatial profiles of Pv (B,E) and the effect of EGTA on release (C,F) showed similar distributions, because Pv and the effect of EGTA were exclusively determined by the [Ca2+]i increase during the step. By contrast, when the step duration was shorter (e.g., 0.5 ms, blue traces), the tail current additionally elevated Pv, and the effect of EGTA decreased. (G) For comparison, release rate traces under the control condition for 0.5 and 5 ms steps with/without tail current are shown. This simulation indicates that the tail current significantly contributes only when the channel is open for a short time. It should be noted that an action potential-induced brief Ca2+ current is mostly mediated by a tail component. [file Image_1.JPEG]
